# Soft tissue sarcoma of the extremities: pending questions on surgery and radiotherapy

**DOI:** 10.1186/s13014-016-0668-9

**Published:** 2016-10-12

**Authors:** Fien Hoefkens, Charlotte Dehandschutter, Johan Somville, Paul Meijnders, Dirk Van Gestel

**Affiliations:** 1 Faculty of Medicine and Health Sciences, University of Antwerp, Antwerp, Belgium; 2 Department of Orthopaedic Surgery, Antwerp University Hospital, Edegem, Belgium; 3 Department of Radiotherapy, University Radiotherapy Antwerp UZA/ZNA, Antwerp, Belgium; 4 Department of Radiotherapy, Institut Jules Bordet, Université Libre de Bruxelles, Brussels, Belgium

**Keywords:** Soft tissue sarcoma, Extremities, Surgery, Radiotherapy

## Abstract

Soft tissue sarcomas are uncommon tumours of mesenchymal origin, most commonly arising in the extremities. Treatment includes surgical resection in combination with radiotherapy. Resection margins are of paramount importance in surgical treatment of soft tissue sarcomas but unambiguous guidelines for ideal margins of resection are still missing as is an uniform guideline on the use of radiotherapy.

The present paper reviews the literature on soft tissue sarcomas of the extremities regarding the required resection margins, the impact of new radiotherapy techniques and the timing of radiotherapy, more particularly if it should be administered before or after surgical resection.

This review was started by searching guidelines in different databases (National Guideline Clearinghouse, EBMPracticeNet, TRIP database, NCCN guidelines,…). After refinement of the query, more specific articles were found using MEDLINE, PubMed, Web of Science and Google Scholar. Used keywords include “soft tissue sarcoma”; “extremities OR limbs”; “radiotherapy”, “surgery”, “margins”, “local recurrence” and “overall survival”. Finally, the articles were selected based on the accessibility of the full text, use of the English language and relevance based on title and abstract.

Literature demonstrates positive resection margins to be an important adverse prognostic factor for local recurrence of soft tissue sarcomas of the extremities. Still, no consensus is reached on the definition of what a good margin might be. The evolution of new radiation techniques, especially Intensity Modulated Radiotherapy, resulted in a s healthy surrounding tissues. However, the timing of radiotherapy treatment remains controversial as both preoperative and postoperative radiotherapy are characterised by several advantages and disadvantages.

## Background

Soft tissue sarcomas (STS) are relatively uncommon tumours, representing 1 % of adult and 7–15 % of paediatric malignancies [1]. In Europe, the incidence is estimated at 4-5/100.000/year [2]. It is a heterogeneous group of tumours of mesenchymal origin that can occur anywhere in the body, with the extremities being the most common primary site, accounting for 60 % of the STS [3, 4]. More than 50 different histological subtypes of STS have been identified. Sarcomas are usually classified into two broad categories: sarcomas of the soft tissues and sarcomas of the bone. Furthermore, STS are subdivided in several subgroups according to localization and treatment (e.g. uterine, extremities, retroperitoneal, etc.) [3, 4]. This paper will be dedicated to STS of the extremities (ESTS).

Up to three decades ago, sarcomas of the extremities were frequently treated with amputation because of the lack of an acceptable alternative concerning local control rates. Advances in treatment, particularly the advent of multimodality treatment, have lowered the amount of amputations needed in the treatment of ESTS and have favoured the use of limb-salvage techniques. In a suboptimal prospective randomized evaluation published in 1982, Rosenberg et al. did not find any differences in overall survival rates or disease-free survival rates when comparing limb-sparing surgery plus RT with amputation [5].

Two randomized series published in the Journal of Clinical Oncology in the nineties proved the importance of adjuvant RT as an important contributor in the success of limb-sparing therapy [6, 7]. In a randomized prospective study of Yang and colleagues postoperative external-beam radiation therapy (EBRT) was shown to decrease the probability of local recurrence in a highly significant way without influencing overall survival rates [6]. Despite several radiation side effects, complaints were mostly transient and few measurable negative effects on quality of Life (QOL) were seen. Pisters et al. found similar results while comparing surgery plus adjuvant brachytherapy with surgery alone [7]. They found adjuvant brachytherapy to improve local control after complete resection of STS. This improvement in local control was limited to patients with high-grade STS. A reduced occurrence of local recurrence in patients with high-grade lesions was not associated with a significant reduction in distant metastasis or improvement in disease-specific survival.

Thus, combination of surgery and RT allows conservation of the limb and function without compromising disease control [4]. In 2003, Clarck et al. found the rate of amputation to be already below 5 % in many oncological centres for patients with primary limb or limb-girdle sarcoma and 9–14 % for recurrent disease [8].

The use of new radiation techniques e.g. intensity modulated radiotherapy (IMRT) has improved QOL and dose localizations, increasing local control and disease-free survival. At the same time a reduction in early and late effects of RT including bone fractures, oedema and joint stiffness was found [6, 9, 10]. The insight into the importance of obtaining a good surgical margin has led to better outcomes, especially for local control, as a positive surgical margin appears to be the most important adverse factor for local recurrence.

The role of chemotherapy in the treatment of STS is still controversial. A meta-analysis by Pervaiz et al. showed only a marginal efficacy with respect to local recurrence, distant recurrence, overall recurrence and overall survival [11].

Patients with large, high grade STS who are at considerable risk for recurrence and metastasis, may benefit from adjuvant chemotherapy [12]. A study of Mahmoud et al. supports the use of neoadjuvant chemotherapy followed by limb-sparing surgery and adjuvant RT for local failure reduction with a trend toward improved disease free survival [13]. However, the role of chemotherapy in the curative setting of high-risk STS remains debatable and its role unclear in the absence of large randomized trials. The increased toxicity and the possibly increased risk of secondary leukaemias also have to be taken into account when delivering chemotherapy [14]. Because unequivocal proof of efficacy is missing, systemic treatment will not be further discussed.

In this paper, the impact of the recent developments in the treatment of ESTS will be studied. For surgery, this includes the optimization of the resection margins. For radiotherapy the focus will mainly be on the IMRT technique and on the differences between preoperative versus postoperative radiotherapy.

## Main text

### Surgery

Different studies have highlighted the importance of adverse prognostic factors in the outcome of patients with ESTS. Age (> 50 years), recurrent disease at presentation and histologic subtypes (e.g. malignant peripheral-nerve tumour) are important factors. But the single most important factor seems to be a microscopically positive margin (R1 resection) [15–18].

In this chapter the importance of surgical margins will be discussed as well as the concept of what a good margin might/should be.

#### I. Surgical procedure

**Fig. 1:**
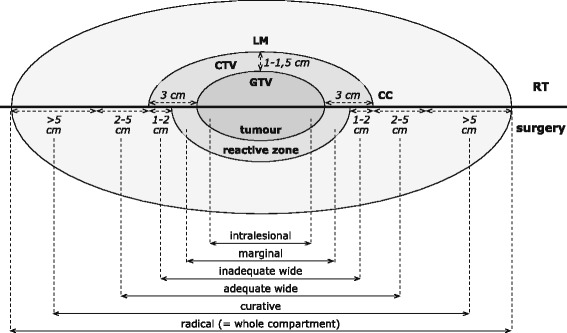
Surgical versus radiotherapeutic margins in the treatment of soft tissue sarcoma of the extremities Schematic description of margins used in the therapy of ESTS. Margins used for radiotherapy differ from those used in surgical resection of the tumour. Nevertheless both include an extra rim of healthy tissue as an attempt to include all microscopic disease around the vast tumour. CC = Cranio-caudal, CTV = clinical target volume, GTV = gross tumour volume, LM = Latero-medial, RT = Radiotherapy

Surgery is the primary treatment for patients with ESTS [18]. Performing surgery, the tumour can be resected with different margins [3, 19]. A *radical* excision is the resection of a full compartment, a *wide* excision is an excision of the tumour with a rim of normal tissue around it and a *marginal* excision is one where the resection margins go through the reactive zone (pseudo-capsule). This reactive zone is a discoloured area around a tumour observable by gross inspection, which is composed of haemorrhagic tissue, scar tissue, degenerated muscle, oedema or tumour capsule. Finally, an *intralesional* resection margin passes through the tumour parenchyma [3, 19]. Kawaguchi et al. define a curative margin as a margin of more than 5 cm outside the reactive zone and a wide margin as a margin of 4–1 cm [19]. Wide margins can be classified into two subgroups: an adequate or an inadequate wide margin. The former is defined as a margin of 2 cm or more and the latter being a margin of 1–2 cm (see Fig. 1).

The standard surgical procedure is a wide excision with negative margins (R0, no residual microscopic disease). To guarantee an R0 resection the cutting face should go through grossly normal tissue planes uncontaminated by tumour. However, it is not always possible to obtain a negative margin and closer margins may be inevitable to preserve critical neurovascular structures or bones. A marginal excision may be acceptable as an individualized option in carefully selected cases. Following the National Comprehensive Cancer Network (NCCN) Guideline 10 mm can be accepted as an adequate margin [3]. A margin of less than 2 mm results in a marginal excision [2]. Because a positive margin is a strong adverse predictor of local recurrence, re-resection must be considered. In the case of R1 resections (i.e. microscopic residual disease) re-resection is an option, but only if adequate margins can be achieved without major morbidity, taking into account tumour extent and tumour biology. In the case of R2 surgery (i.e. gross residual disease), re-resection in a reference centre is mandatory, possibly with preoperative treatments if adequate margins cannot be achieved, or when surgery is thought to be mutilating. If re-resection is not possible or positive margins remain present after re-resection post-operative RT is indicated [2, 3].

The NCCN guidelines also provide instructions on the treatment of STS depending on tumour stage [3]. Using the American Joint Committee on Cancer (AJCC) staging system (see Table 1; [20]) the NCCN guidelines recommend to proceed as follows:

**Table 1:** Definitions and Staging System of the American Joint Committee on Cancer, 7th Edition [74]

	Primary tumour	Regional Lymph Nodes	Distant metastasis	Grade^a^
Stage IA	T1a	N0	M0	G1, GX
	T1b	N0	M0	G1, GX
Stage IB	T2a	N0	M0	G1, GX
	T2b	N0	M0	G1, GX
Stage IIA	T1a	N0	M0	G2, G3
	T1b	N0	M0	G2, G3
Stage IIB	T2a	N0	M0	G2
	T2b	N0	M0	G2
Stage III	T2a, T2b	N0	M0	G3
	Any T	N1	M0	Any G
Stage IV	Any T	Any N	M1	Any G

Primary Tumour (T)

TX, Primary tumour cannot be assessed

T0, No evidence of primary tumour

T1, Tumour 5 cm or less in greatest dimension*

-T1a, Superficial tumour

-T1b, Deep tumour

T2, Tumour larger than 5 cm in greatest dimension*

-T2a, Superficial tumour

-T2b, Deep tumour

*Note: Superficial tumour is located exclusively above the superficial fascia without invasion of the fascia; deep tumour is located either exclusively beneath the superficial fascia, superficial to the fascia with invasion of or through the fascia, or both superficial to and beneath the fascia

Regional Lymph Nodes (N)

NX, Regional lymph nodes cannot be assessed

N0, No regional lymph node metastasis

N1**, Regional lymph node metastasis

**Note: Presence of positive nodes (N1) in M0 tumours is considered Stage III

Distant Metastasis (M)

M0, No distant metastasis

M1, Distant metastasis

^a^See Table 2 for explanation FNCLCC grading system

##### Stage I

Stage I STS should be treated with surgery to obtain adequate surgical margins. If such margins can be obtained or there is an intact fascial plane there is a sufficient long-term local control. When appropriate margins cannot be obtained, RT needs to be considered. However for stage IA tumours (< 5 cm) a wait and see attitude can be adopted.

##### Stage II and III, resectable disease with acceptable functional outcomes

Stage IIA STS can be treated with surgery only in case of small tumours when resection with wide margins is possible, otherwise pre- or postoperative radiotherapy can be delivered in addition to surgery.

In stage IIB or III tumours, surgery to obtain appropriate surgical margins is combined with radiotherapy (pre- or postoperatively).

##### Stage II and III, resectable disease with adverse functional outcomes or unresectable primary disease

When the tumour cannot be resected with adequate margins, radiotherapy should be given preoperatively to downstage the tumour and to enable effective surgical resection. When the mass becomes resectable it should be resected to obtain appropriate surgical margins again followed by RT. If the tumour remains unresectable the further options are definitive RT, chemotherapy, palliative surgery, observation (if asymptomatic), sufficient supportive care and amputation.

However, the question about the appropriate surgical margin for satisfactory local control remains unanswered so far.

#### II. Factors influencing choice of margin

The adequate minimal margin may depend on several factors, including histological subtype, adjuvant therapies and the presence of resistant anatomical barriers, such as muscular fasciae, periosteum and epineurium [2–4].

##### Histological subtype

The NCCN guidelines recommend resection margins based on the malignancy grade determined following the French Federation on Cancer Centres Sarcoma Group (FNLCC) grading system [3, 21] (see Table 2).

**Table 2:** Definitions of Grading Parameters for the FNCLCC System [21]

Parameter	Criterion
Tumour Differentiation	
Score 1	Sarcoma closely resembling normal adult mesenchymal tissue (e.g., well-differentiated liposarcoma)
Score 2	Sarcomas for which histologic typing is certain (e.g. myxoid liposarcoma)
Score 3	Embryonal and undifferentiated sarcomas; sarcomas of uncertain type
Mitosis Count	
Score 1	0-9/10 HPF
Score 2	10-19/10 HPF
Score 3	≥20/10 HPF
Tumour Necrosis (Microscopic)	
Score 0	No necrosis
Score 1	≤50 % tumour necrosis
Score 2	>50 % tumour necrosis
Histologic Grade	
Grade 1	Total score 2,3
Grade 2	Total score 4,5
Grade 3	Total score 6,7,8

*FNCLCC* Fédération Nationale de Centres de Lutte Contre le Cancer, *HPF* high-power field

E.g., the cutaneous leiomyosarcoma is a primarily low-grade malignancy. Deneve et al. concluded that good oncological control and excellent outcomes are possible with an only 1-cm resection margin in most cases [22]. This is in contrast with the treatment of the aggressive cutaneous angiosarcoma consisting of surgical excision with wide margins often combined with radiotherapy. Moreover, in some cases even amputation remains unavoidable [23].

##### Adjuvant therapies

Pre- and postoperative RT can influence surgical margins. As stated above the combination of surgery and RT allows conservation of the limb and function without compromising disease control, drastically lowering the need for amputation [4, 8].

RT biologically “sterilizes” microscopic extensions of tumour thereby limiting the need for extensive margins, consequently allowing sparing of critical organs and neurovascular bundles [24]. Another possibly favourable aspect of preoperative RT is the potential reduction of the seeding of microscopic sarcomatous cells at the time of surgical resection in addition to tumour shrinkage itself, which will greatly enhance the ability of the surgeon to achieve negative margins [25].

Different aspects of RT in the treatment of ESTS will be discussed later in this paper.

##### Resistant anatomical barriers

The word ‘barrier’ refers to any tissue that has resistance against tumour invasion, and can include muscle fascia, joint capsule, tendon, tendon sheath, epineurium, vascular sheath, cartilage, pleura and peritoneum. Barriers can be classified as either being a thick or a thin barrier. A thick barrier is a physically strong membranous tissue with a white tendinous luster, e.g. an iliotibial band, a presacral fascia or a joint capsule. The periosteum of an infant or young child can also be included in this category. A thin barrier is a weaker membranous tissue e.g. muscle fascia, the periosteum of an adult, vessel sheath or epineurium [19].

Kawaguchi et al. tried to find a solution for the lack of information about an appropriate surgical margin in the staging system of Enneking [19, 26]. The surgical staging system of Enneking is helpful in creating effective communication between different institutions all over the world and provides a uniform language for comparison of treatment [26]. However it does not solve the problem on how to choose a good margin balancing between local control of the lesion and maximum preservation of function. Therefore, Kawaguchi et al. developed a modification of the system of Enneking for better evaluation of the surgical margins and for better refinement of limb salvage surgery [19]. By considering barrier effects translated into concrete distance equivalents, surgery can be done at sites where barriers exist by using a smaller margin than true physical distance. It then becomes possible to choose a surgical plan more safely. To clarify the minimal margin when barriers exist, tumour excision with extremely small margins, which nonetheless established local cure, were accumulated and their barriers were considered to be the equivalent of 5 cm thick healthy tissue. To convert these least safe margins into a 5 cm equivalent distance, particular scores were assigned to each type of barrier, with the final scores being decided through several assessments of the patients.

Until now, conservative surgery with wide resection, having 1 cm of normal tissue margin or intact anatomical barriers, such as muscular fascia or periosteum, is the most commonly performed procedure [27]. The European Society for Medical Oncology (ESMO) guidelines also tried to define the anatomical structures that could be used as good barriers for surgery [2]. They concluded that muscular fascia, periosteum and epineurium are resistant anatomical barriers.

Therefore, in the presence of an anatomical structure functioning as a resistant barrier for tumour growth, smaller resection margins can be acceptable [2, 19, 27, 28].

#### III. Impact of surgical margin on outcome

##### Local control

There is strong evidence that a positive surgical margin is a strong predictor of local recurrence for patients with ESTS. The status of the surgical margin is the factor having the most profound effect on local recurrence as reported consistently in literature [16, 28–31].

In a prospective study of 1041 patients with ESTS Pisters et al. found a microscopically positive surgical margin to be a significant adverse prognostic factor for local recurrence [15]. Of the 1041 patients that were followed 242 (23 %) had a positive surgical margin. Of these, 64 (26 %) developed local recurrence. The study of Pister et al. was also the first study to suggest that there is a relationship between positive margins and tumour-related mortality. They found that the adverse prognostic factors for local recurrence in ESTS were different from those that predict distant metastasis and disease free survival. This has a clinical implication because staging systems to stratify patients for risks of distant metastasis and tumour-related mortality using these prognostic factors will not stratify patients for local recurrence.

Many years later, in 2012 David J. Biau et al. performed a similar study [32]. In their cohort of 1668 patients with a STS of the extremity or trunk patients with positive surgical margins had a 3.3 times greater risk of developing a local recurrence compared with those who had negative surgical margins.

##### Distant recurrence and metastasis

A lot of uncertainties remain on the impact of surgical margins on distant metastasis. It is most likely that the surgical margins do not have a direct influence on distant metastasis. Local recurrence is associated with an increased risk of metastasis but an inadequate surgical margin is not a risk factor for metastasis. The study of Trovik et al. confirms, in regard to metastasis, tumour-related risk factors (malignancy grade and tumour size) to be much more important than treatment-related risk factors like surgical margins [16]. Local recurrence was associated with an increased metastasis rate, whereas inadequate surgical margin was a risk factor for local recurrence but not for metastasis. Other studies came to the same conclusions [33–35]. Deep seated lesions, tumour size > 10 cm, high-grade and recurrence after radical surgery are all found to be high risks factors for distant metastasis [31].

##### Overall survival

The effect of surgical margin and local recurrence on overall survival is a well-discussed issue in literature as local recurrence is a significant factor associated with decreased survival [17, 35, 36]. Patients with positive surgical margins, putting them at high risk for local failure, should be considered for increased surveillance, as local failure is associated with subsequent metastasis and decreased survival [35]. This association between local failure and decreased survival makes it very important to achieve negative resection margins in STS. If this is not achieved after primary treatment a re-excision is recommended. Lack of re-excision after local failure has proven to be associated with a decreased overall survival and an increased risk of metastasis as found in the study of Zagars et al. [37].

Newer evidence found unplanned resection at a non-referral hospital to be the most important risk factor for overall survival because of the high rates of inappropriate margins achieved in those cases. Accurate diagnosis and adequate initial surgery are the most important factors for improving clinical outcomes.

Treatment in specialized sarcoma centres is therefore crucial and can be life-saving [38].

In conclusion, the importance of good surgical margins after surgery of ESTS in achieving local control has been proven in different studies [31, 39–41]. However the relationship between surgical margins and distant metastasis remains unclear [16].

Inadequate surgical margins, local recurrence and metastasis are all significantly associated with decreased overall survival [30]. However, although appropriate margins are associated with a better prognosis, there still is no general agreement on the definition of the ideal margin [24].

### Radiotherapy

Radiotherapy is an essential adjunct to surgery for adult soft-tissue sarcomas in optimizing both local control and functional outcome [42]. According to the NCCN guidelines RT should always be implemented in the therapy of STS in stage II or III disease [3]. In stage I STS RT can be considered when resection margins are less than 1 cm in the absence of intact fascial planes. RT can be applied neoadjuvant (preoperative) or adjuvant (postoperative) to surgery. The advantages and disadvantages of both approaches will be discussed later in this paper. Exceptionally, radiotherapy can also be administered as a primary local therapy when general health is too poor to undergo surgery or when the sarcoma cannot be curatively removed e.g. when it has already spread [9, 43]. Outcomes in patients with STS have improved with new developments in RT technology such as IMRT and intraoperative radiation therapy (IORT) [9].

Despite its proven efficacy a recent article of Bagaria et al. reports an underuse of RT for a significant amount of STS patients in the US [44]. More effort needs to be directed towards compliance since only 60 % of the stage II and III tumours underwent RT whereas 25 % of the stage I tumours did, even though not recommended for this group.

This section will give a brief overview of the different RT-techniques.

#### I. Types of radiation therapy

##### External radiation

*External radiation* is the most commonly used form of RT [43]. A very important evolution in external beam RT is the development of *intensity modulated RT* (IMRT) since the late nineties. The main advantage of this technique is its sharp dose gradients enabling very precise irradiation of the target volumes, thereby minimizing the high dose radiation to the surrounding healthy tissues [45]. This makes IMRT especially suitable for treating complex treatment volumes minimizing dose to organs at risk (OAR) nearby, that otherwise might necessitate dose limitations [42].

The past two decades, IMRT became a standard technique despite its drawbacks of volume delineation, planning, robustness of delivery, challenging quality assurance and cost as compared with non-IMRT. Theoretically, the advantages of IMRT over non-IMRT are well accepted but insufficient evidence is available to conclude for the clinical setting. The main incentive to choose IMRT over non-IMRT is its capacity to reduce toxicity. Findings regarding survival, tumour control or other indexes of treatment efficacy remain generally inconclusive. Comparative case series show no differences in disease control and survival unless dose escalation was used [46].

In the study of Alektiar and colleagues, the low risk of complications was confirmed [10]. Even in a population at high risk for bone fractures only 4,8 % actually developed a fracture. To treat these fractures, no surgical intervention was required, which is unusual with RT-associated bone fractures. Other complications such as oedema and joint stiffness also decreased when compared with conventional RT. Despite the excellent results of adjuvant IMRT for primary ESTS in this study, more investigations are needed to confirm the data on a larger number of patients and with a longer period of follow-up.

In 2013 two prospective phase II studies, which compared IMRT with classical RT in its rate of normal tissue morbidity, have been published. O’Sullivan et al. found a numerical reduction in wound complications while using preoperative image-guided IMRT compared to classical RT [47]. This reduction in wound complications however, did not reach statistical significance, but it did significantly diminish the need for tissue transfer. Chronic RT morbidities and the need for subsequent secondary operations for wound complications were lowered, although not significantly, whereas good limb function was maintained. Wang and colleagues, on the other hand, showed IMRT to significantly reduce the RT related late morbidities as fibrosis, oedema and joint stiffness [48]. IMRT provided excellent local control for patients with STS of the extremities and trunk. In a recent study, Folkert et al. even showed a significant reduction in local recurrences when comparing IMRT and conventional RT in treatment of primary ESTS [49]. In this single institution study 319 patients with primary ESTS were treated using limb-sparing surgery and adjuvant RT, 154 patients using classical external beam RT and 165 patients using IMRT. On multivariable analysis adjusting for patient age and tumour size, IMRT retained significance as an independent predictor of reduced local recurrence.

A possible concern about IMRT is that its tight dose distribution, an advantage in reducing RT morbidity to surrounding normal structures, might compromise tumour coverage. However, Alektiar and colleagues showed, in a group of high-risk patients, IMRT to contribute to an excellent local control [10]. In some instances the tumour coverage may even be improved using IMRT. For example with large thigh sarcomas, the limb contour near the groin is significantly different from the contour near the knee, leading to underdosage, with a potential local relapse, in the former and overdosage, a potential for toxicity, in the latter in the case of conventional RT. This is no longer a problem when using IMRT.

Moreover, recently detailed guidelines for RT target volume delineation have been published by the Radiation Therapy Oncology Group (RTOG; preoperative RT) and by Haas and his European, American and Canadian colleagues (pre- and postoperative RT) [50, 51].

For preoperative RT of primary large high-grade ESTS the gross tumour volume (GTV), being the volume of known infiltration, is defined by T1 contrast-enhanced magnetic resonance images according to the RTOG [50]. The clinical target volume (CTV), being the volume of suspected (microscopic) infiltration, is defined as the GTV plus 3 cm margins in the longitudinal directions, limited to the compartment. The radial margin from the lesion should be 1–1.5 cm if not confined by intact fascial barrier, bone or skin surface. This CTV should be manually edited to encompass any suspicious oedema on MRI T2 images. Haas & al. confirm these definitions except for the CTV in the longitudinal direction, which is defined as the GTV plus 4 cm margins, limited to the compartment [51]. A dose of 50 Gy in 25 fractions should be administered to this target volume [3, 51, 52] (See Fig. 1). In function of the resection margins, an additional ‘boost’ dose of 16–26 Gy to the high-risk zone can be indicated (see post-operative RT).

Therewithal they published guidelines for RT target volume delineation in postoperative RT. The GTV cannot be defined after removal of the tumour but the original tumour extensions should be recreated within the planning CT data set. The first a portion of the dose (45–50.4 Gy) is applied to a larger volume encompassing the surgical bed with appropriately safety margins, called the elective CTV. This is followed by supplementary dose (16–26 Gy) to a smaller ‘boost’ CTV [3, 51]. The elective CTV is defined as the reconstructed initial tumour volume surrounded by the same margins as used for preoperative RT. However, this volume might have to be adjusted for it needs to include all visible clips, drain sites, the entire length of the scar and the entire extent of the operative field. The boost CTV is the same volume as the elective CTV, except in the longitudinal direction, where it is defined by the reconstructed GTV plus only a 2 cm margin.

##### Intraoperative radiation therapy (IORT)

IORT is one of the advances in therapy of STS. One large dose of radiation is administered during surgery, after resection but before stitching the wound. Hereby the operative field can be irradiated directly and the healthy tissues can be spared. This type of RT is often combined with postoperative RT [43]. IORT is mainly used in treatment of STS in the pelvis or abdomen, its use in peripheral tumours is limited although a study of Tran et al. in 2006 showed that IORT used as a boost to EBRT provides excellent local control, with limited acute toxicities when used in treating ESTS [4]. These findings were confirmed in a recent study of Call et al. on upper-extremity STS where treatment including IORT was associated with excellent local control, limb preservation and survival [53].

##### Brachytherapy

Sometimes called internal radiation therapy, brachytherapy involves the direct application of radioactive sources into the tumour bed through catheters placed during surgery. With a fast decay of the dose by the distance, these sources are able to deliver a highly concentrated radiation dose more conformal compared to external beam RT, resulting in improvement in local control together with better sparing of the surrounding healthy tissues. Two different types of brachytherapy can be distinguished: high-dose rate (HDR) brachytherapy and low-dose rate (LDR) brachytherapy. In HDR brachytherapy, a great amount of radiation is emitted over a short period of time and the sources stay in place for only a few minutes. For the LDR technique the sources may stay in place for several days [43]. LDR and HDR brachytherapy are associated with similar rates of local control. It has been suggested that HDR brachytherapy may be associated with lower incidences of severe toxicity. However, this has not been proven in randomized clinical trials [3, 54]. Pulsed Dose Rate (PDR) brachytherapy is a relatively new RT modality. It uses stronger radiation sources compared to LDR brachytherapy but simulates its total dose by providing pulses of 10 to 30 minutes long exposures every hour. It thereby combines physical advantages of HDR technology with radiobiological advantages of LDR brachytherapy [55].

Brachytherapy is mostly administered as a boost that is supplemented with EBRT, but it can also be an attractive alternative to EBRT as a form of adjuvant RT. The period of treatment is much shorter, the financial costs are lower, evaluation at the time of surgery is possible and by sparing the surrounding tissues needless complications can be averted [56, 57]. However, brachytherapy techniques require special expertise and experience, restricting its availability.

##### Hadron therapy

With hadron therapy charged particles (protons and other ions such as carbon) are used to irradiate the tumour. A recent advancement in therapy of STS is *Proton-Beam RT* where a beam of protons irradiates the tumour. Compared to photons (e.g. x-rays), protons allow a superior dose distribution because protons depose little energy in tissue until near the end of the proton range where the residual energy is deposed over a short distance resulting in a steep peak in the absorbed dose known as the Bragg peak. Despite this advantage it has not been proven to be a better treatment in STS patients and its availability is limited [9, 43]. *Heavy-ion therapy* is the use of particles more massive than protons or neutrons, such as carbon ions. Compared to protons, carbon ions are correlated with a higher density of ionization at the end of their range increasing the biological efficiency of the dose and making them less dependent of oxygen and therefore interesting in the treatment of radioresistant and hypoxic tumours. Notwithstanding this advantage, the costs of this form of RT are very high restricting its availability to less than ten facilities worldwide [58].

In conclusion, advances in radiotherapy techniques diminish normal tissue complications because of their dosimetric advantages compared to conventional RT and might result in higher local control rates.

#### II. Timing

There is still a lot of debate whether RT should be given before or after surgery and what the best interval between surgery and RT can/should be? A tendency arises to choose for the preoperative technique. In the USA this type of RT is predominant over the post-operative form. In Europe the post-operative type remains the mainstream. Next, an overview of the primordial advantages and disadvantages of both techniques will be given.

##### Preoperative versus Postoperative RT (see Table 3)

**Table 3:** Advantages and disadvantages of pre- and postoperative radiotherapy

	Advantages	Disadvantages
Preoperative Radiotherapy	• Smaller RT volume • Easier resection • Better oxygenations and vascularization of the area = > larger effect • Reduction of late complications	• Wound complications • Postponing surgery • Fibrosis that could hamper surgery
Postoperative Radiotherapy	• Better staging • Less scar complications	• Large RT Volumes • More late complications (fibrosis, joint stiffness, oedema) • Need for demarcation of the operation field (clips)

***Preoperative RT***

Preoperative RT has several *advantages*. First, the treatment volume can be smaller because there is no need to cover the entire operative field, resulting in a lower integral dose. The use of preoperative RT can thereby reduce the risk for late complications [59]. In a study of Nielsen et al., the field size used for preoperative irradiation was compared with the field size needed to treat that same patient postoperatively [60]. Preoperatively a radial margin of 5 cm around the tumour was used for low and intermediate grade and 7 cm for high-grade sarcomas. The same margins were used to treat the tumour postoperatively but now around the surgical field. Independently of surgical procedure and tumour grade, the size of the preoperative radiation field and the number of joints included in the field were significantly smaller than in the postoperative radiation setting. Stated that the rate of complications is proportional to the magnitude of the radiated area and when the same radiation parameters regarding time, dose and fraction are used, a lower incidence of late complications may be expected with preoperative RT [59, 60].

Furthermore, resection of a tumour can become easier after administration of preoperative RT. The tumour may or may not regress with preoperative RT, but the pseudocapsule may thicken and become acellular, easing resection and decreasing the risk of recurrence [3]. At last, by applying RT before surgery, better oxygenation and vascularization of the area ensures a greater effect of RT, permitting a lower total dose to be administered, resulting in a better functional outcome.

However, preoperative RT has also several *disadvantages*. The main disadvantage is the frequent occurrence of major wound complications, especially in the lower limbs. These complications can have a detrimental effect on the function of STS patients [61, 62]. In a randomized trial, O’Sullivan et al. compared the appearance of wound complications in preoperative and postoperative RT [59]. The primary endpoint was rate of wound complications within 120 days of surgery. Wound complications were recorded in 35 % and 17 % of the preoperative and postoperative group, respectively. Overall survival was slightly better in patients who received preoperative RT. Another important disadvantage is the delay in surgery. After RT, an interval of 3–6 weeks is required to decrease the risk of wound complications and to cool down the acute reactions. Nevertheless, it is not recommended either to create an interval that is too long because this can lead to the development of late fibrosis which can hamper surgery [3]. However, through the advances in RT mentioned above, this inconvenient side effect has been reduced by avoiding high dose radiation on the healthy surrounding tissues.

If a wide resection is possible, additional RT may be redundant. However if the tumour is located nearby a neurovascular bundle or next to bony structures, close margins may be unavoidable. In this case a postoperative RT boost with brachytherapy, IORT or EBRT is recommended [63]. The usual dose of preoperative RT is 50 Gy. For the postoperative boost different dose levels exist. The NCCN guidelines recommend doses of 10–14 Gy for close margins, 16–20 Gy for microscopically positive margins (R1), and 20–26 Gy for grossly positive margins (R2) [3]. However, some data suggest that some patients with positive margins following preoperative RT and surgery may do well without a boost [64, 65]. Ali Al Yami et al., found no difference in local control comparing patients with a margin-positive excision who received preoperative RT alone with patients who received preoperative RT and a postoperative boost [64]. Moreover, higher radiation doses contain a greater risk for late complications such as fractures, fibrosis, oedema and joint stiffness. The recent study of Alamanda et al. confirmed these findings [65]. No differences in rates of local recurrence, distant metastasis or death due to STS were found in patients who received a postoperative boost versus those who did not.

***Postoperative RT***

To eliminate any cancer cells that may remain after surgery, RT can be given postoperatively as an adjuvant therapy. Also postoperative RT is characterized by several advantages and disadvantages.

When a tumour is resected before radiotherapy, better staging of the tumour is possible. The amount of scar complications is also lower. The number of late complications, on the other hand, rises. A study of Davis and colleagues showed significantly more late fibrosis in postoperative RT compared to preoperative RT [66]. Although not statistically significant, the percentage of patients with late oedema and joint stiffness was also higher in the postoperative group. The higher rate of late complications compared to preoperative RT may be due to the higher total radiation dose, 50 Gy preoperatively (no boost necessary when negative margins are reached) compared to 60–66 Gy in postoperative RT, and the larger area that needs to be irradiated [3]. A last possible difficulty concerning postoperative RT lies in the need for optimal cooperation between radiotherapist and surgeon. The surgeon plays a major role in the RT success rate by marking the operation field with clips to indicate the area that needs to be irradiated.

After surgery RT can be administered intra-operatively, with brachytherapy or by EB RT. Most of the times the entire surgical site is included. The total dose should always be determined by the tolerance of the healthy tissues. According to the NCCN guidelines, a boost of 16–18 Gy should be given if microscopical residual tumour (R1) is left behind. In case of gross residual disease (R2), a boost of 20–26 Gy is indicated [3]. Decisions regarding the use of postoperative RT should always be individualized and should not only be based on positive or negative resection margins as described above. Histological grade, age of the patient, localisation of the tumour including vicinity of neurovascular structures etc. should be taken into account.

Adjuvant RT has been shown to improve local control in ESTS. Alektiar et al. showed adjuvant RT to improve local control in patients with high-grade ESTS with positive margins [67]. In a more recent report of 2008 Jebsen and colleagues also showed that adjuvant RT effectively prevents local recurrences in STS, irrespective of the tumour depth, malignancy grade and surgical margin status [68].

According to a paper of the International Journal of Radiation Oncology, the interval between surgery and postoperative RT does not significantly impact the 10-year local control rate [69]. Hence, a RT delay should not be viewed as an independent adverse factor for local control and therefore it should not be compensated with increased doses RT.

Regardless of all the differences in advantages and disadvantages in the use of preoperative or postoperative RT, multiple studies did not find evidence for differences in disease outcome [59, 70–73].

## Conclusions

In the last decades, multi-modality treatment has improved functional outcome of ESTS. However, a lot of questions remain.

Regarding *surgery*, the importance of surgical resection margins can no longer be underestimated. Literature has reached consensus on the adverse effect of positive resection margins on local control of STS. Positive margins and local recurrence also seem to have an important influence on overall survival. However the concept of an ideal surgical margin remains unclear. There is a high need for standardisation and guidelines concerning good surgical margins for ESTS resection.

*Radiotherapy* improves local control of ESTS compared with surgery alone. New techniques as IMRT and IORT, although technically challenging, are implemented in the treatment of STS because of their dosimetric advantages compared to conventional RT. Healthy tissues are spared and the amount of normal tissue complications diminishes. Especially the introduction of IMRT in the last 20 years has resulted in an important decrease in toxicity. Pre- and postoperative RT each have their own advantages and disadvantages. It is, however, not clear which RT sequence is superior.

For surgery as well as radiotherapy, further research is necessary and urgent.

## Abbreviations

AJCC: American Joint Committee on Cancer
CT: Computed tomography
CTV: Clinical target volume
EBRT: External beam radiation therapy
ESMO: European Society for Medical Oncology
ESTS: Extremity soft tissue sarcomas
FNLCC: French Federation on Cancer Centres Sarcoma Group
GTV: Gross tumour volume
Gy: Gray
HDR: High dose rate
IMRT: Intensity modulated radiotherapy
IORT: Intraoperative radiation therapy
LDR: Low dose rate
NCCN: national comprehensive cancer network
OAR: Organs at risk
PDR: Pulsed dose rate
QOL: Quality of life
RT: Radiotherapy
RTOG: Radiation Therapy Oncology Group
STS: Soft tissue sarcomas
